# Fast electrosynthesis of Fe-containing layered double hydroxide arrays toward highly efficient electrocatalytic oxidation reactions†
†Electronic supplementary information (ESI) available. See DOI: 10.1039/c5sc02417j


**DOI:** 10.1039/c5sc02417j

**Published:** 2015-08-12

**Authors:** Zhenhua Li, Mingfei Shao, Hongli An, Zixuan Wang, Simin Xu, Min Wei, David G. Evans, Xue Duan

**Affiliations:** a State Key Laboratory of Chemical Resource Engineering , Beijing University of Chemical Technology , Beijing 100029 , China . Email: shaomf@mail.buct.edu.cn ; Email: weimin@mail.buct.edu.cn ; Fax: +86-10-64425358 ; Tel: +86-10-64412131

## Abstract

A new electrochemical synthesis route was developed for the fabrication of Fe-containing layered double hydroxide (MFe-LDHs, M = Ni, Co and Li) hierarchical nanoarrays, which exhibit highly-efficient electrocatalytic performances for the oxidation reactions of several small molecules (water, hydrazine, methanol and ethanol). Ultrathin MFe-LDH nanoplatelets (200–300 nm in lateral length; 8–12 nm in thickness) perpendicular to the substrate surface are directly prepared within hundreds of seconds (<300 s) under cathodic potential. The as-obtained NiFe-LDH nanoplatelet arrays display promising behavior in the oxygen evolution reaction (OER), giving rise to a rather low overpotential (0.224 V) at 10.0 mA cm^–2^ with largely enhanced stability, much superior to previously reported electro-oxidation catalysts as well as the state-of-the-art Ir/C catalyst. Furthermore, the MFe-LDH nanoplatelet arrays can also efficiently catalyze several other fuel molecules’ oxidation (*e.g.*, hydrazine, methanol and ethanol), delivering a satisfactory electrocatalytic activity and a high operation stability. In particular, this preparation method of Fe-containing LDHs is amenable to fast, effective and large-scale production, and shows promising applications in water splitting, fuel cells and other clean energy devices.

## Abstract 2

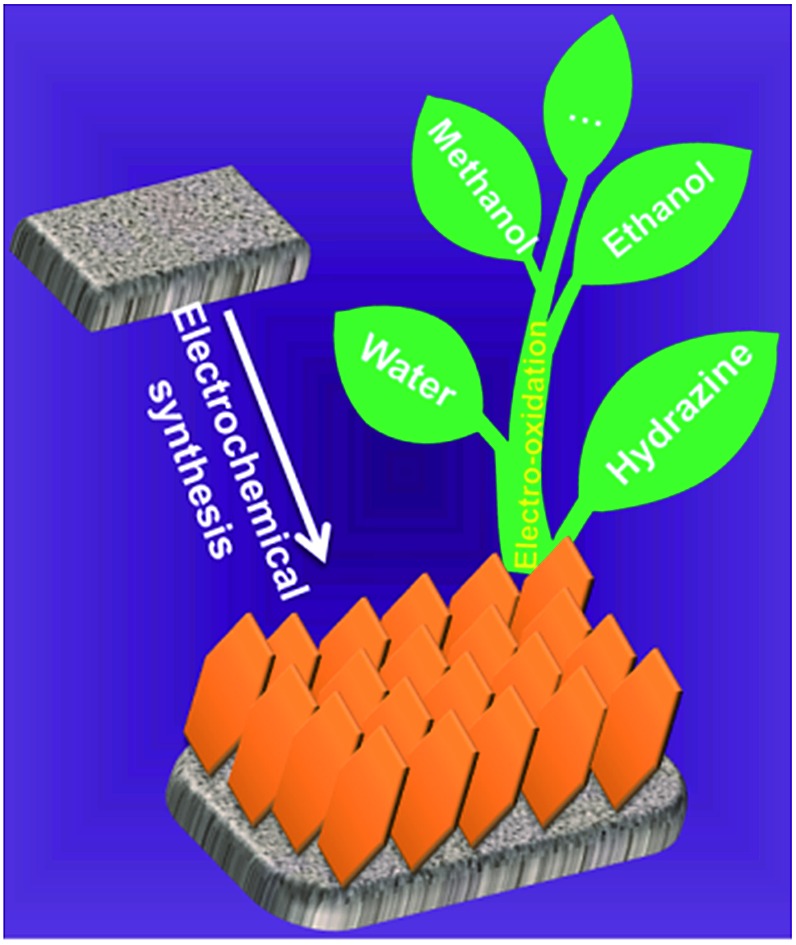
Fast electrosynthesis of Fe-containing layered double hydroxide arrays and their highly-efficient electrocatalytic performance toward small molecule oxidation reactions.

## Introduction

Small molecule electro-oxidation reactions (*e.g.*, water, hydrazine, methanol or ethanol), as the core processes of water splitting devices, metal–air batteries or fuel cells, have attracted considerable attention owing to the increasing demands for renewable energy resources.^1^–^8^ In practice, however, anodic electro-oxidation processes are greatly constrained by high kinetic barriers (high overpotentials), sluggish reaction dynamics and instability of the electrode materials. For instance, even in the presence of state-of-the-art precious metal catalysts (such as Pt,^9^,^10^ Pd,^11^–^13^ IrO_2_ (ref. 14) and RuO_2_ (ref. 15)), a substantial overpotential is still required to drive the oxidation of small molecules such as hydrazine, ethanol and water. Moreover, noble metal catalysts are also disadvantageous because of their scarcity, high cost and high toxicity. Recently, great efforts have been focused on the oxides/hydroxides of first-row transition metals as promising electro-oxidation catalysts. Among them, cobalt-based composites,^16^–^19^ perovskite oxides^20^–^22^ and oxyhydroxides (*e.g.*, amorphous FeOOH and NiOOH)^23^–^25^ were extensively studied and have shown interesting catalytic behavior. Despite all this progress, the development of stable, efficient and cost-effective electro-oxidation catalysts toward small molecules still remains a challenge.

Layered double hydroxides (LDHs) are a large class of typical inorganic layered materials which can be described by the general formula [M_1–*x*_^II^M_*x*_^III^(OH)_2_]^*z*+^(A^*n*–^)_*z*/*n*_·*y*H_2_O (M^II^ and M^III^ are divalent and trivalent metals, respectively; A^*n*–^ is the interlayer anion compensating for the positive charge of the brucite-like layers).^26^–^31^ Recently, LDH materials have been found to show surprising oxygen evolution reaction (OER) performances and gained intensive attention as water oxidation catalysts.^32^–^45^ For instance, Gong and coworkers reported that NiFe-LDH shows higher electrocatalytic activity and stability for the OER in alkaline environments than commercial precious metal-based catalysts.^36^ Subsequently, various nanostructures of NiFe-LDHs (*e.g.*, 2D single-layer nanoplatelets^37^ and 3D architectural films^38^) as well as their nanocomposites (*e.g.*, NiFe-LDHs/graphene,^39^–^42^ and NiFe-LDHs/carbon quantum dot^43^) with excellent OER performances have been further studied. Although these results indicate that NiFe-LDH materials serve as rather good OER catalysts, time- and cost-effective synthesis methods with good control over the hierarchical nanostructures are highly necessary for further exploration of LDH-based electrodes with enhanced properties. In addition, the generalized electrocatalytic oxidation properties of Fe-containing LDHs toward other small fuel molecules (*e.g.*, hydrazine, methanol and ethanol) remain unknown. In the past few decades, some advances have been made to find facile ways of preparing LDH materials (*e.g.*, electrosynthesis method) with the merits of a fast and one-pot synthesis on the electrode surface.^46^–^48^ However, the development of fast and generally applicable methods for Fe-containing LDHs, so as to achieve excellent electrocatalysts for small molecule oxidation reactions, is still a challenge in terms of both scientific and technological considerations.

Herein, we demonstrate the electrochemical approach as a fast, precisely controllable and economic method to fabricate various Fe-containing LDH hierarchical nanoarrays for efficient electrocatalytic oxidation reactions. Homogeneous and uniform LDH nanoplatelets anchoring onto the surface of the conducting substrates can be accomplished at room temperature within hundreds of seconds. Remarkably, the NiFe-LDH nanoplatelet arrays exhibit optimal activity and long-term durability for water oxidation, in comparison with other electrocatalytic materials reported to date. Moreover, the universality of the electrocatalysis of the NiFe-LDH arrays toward other small molecule oxidations in fuel cells (N_2_H_4_, CH_3_OH and C_2_H_5_OH) has also been demonstrated. This time- and cost-effective synthesis route holds great promise for large-scale industrial manufacture, and is expected to show promising applications in renewable energy resources.

## Results and discussion

### Structural and morphological characterization of MFe-LDH

**Fig. 1:**
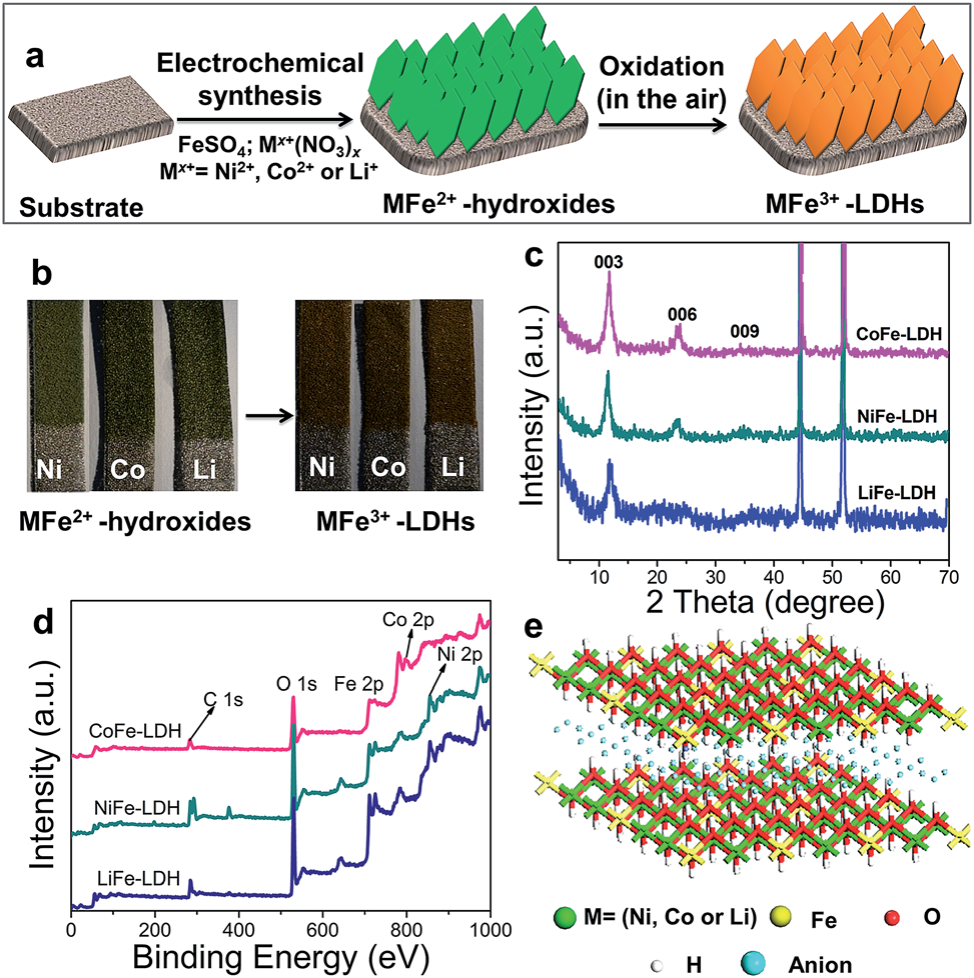
(a) Scheme of the synthetic route to the MFe-LDH (M = Co, Ni and Li) nanoarrays; (b) photograph of the MFe-LDH nanoarrays growing on the Ni foam before and after self-oxidation in the air; (c) XRD patterns and (d) XPS spectra of the MFe-LDH (M = Ni, Co and Li) nanoplatelet arrays; (e) structure model of MFe-LDH.

**Fig. 2:**
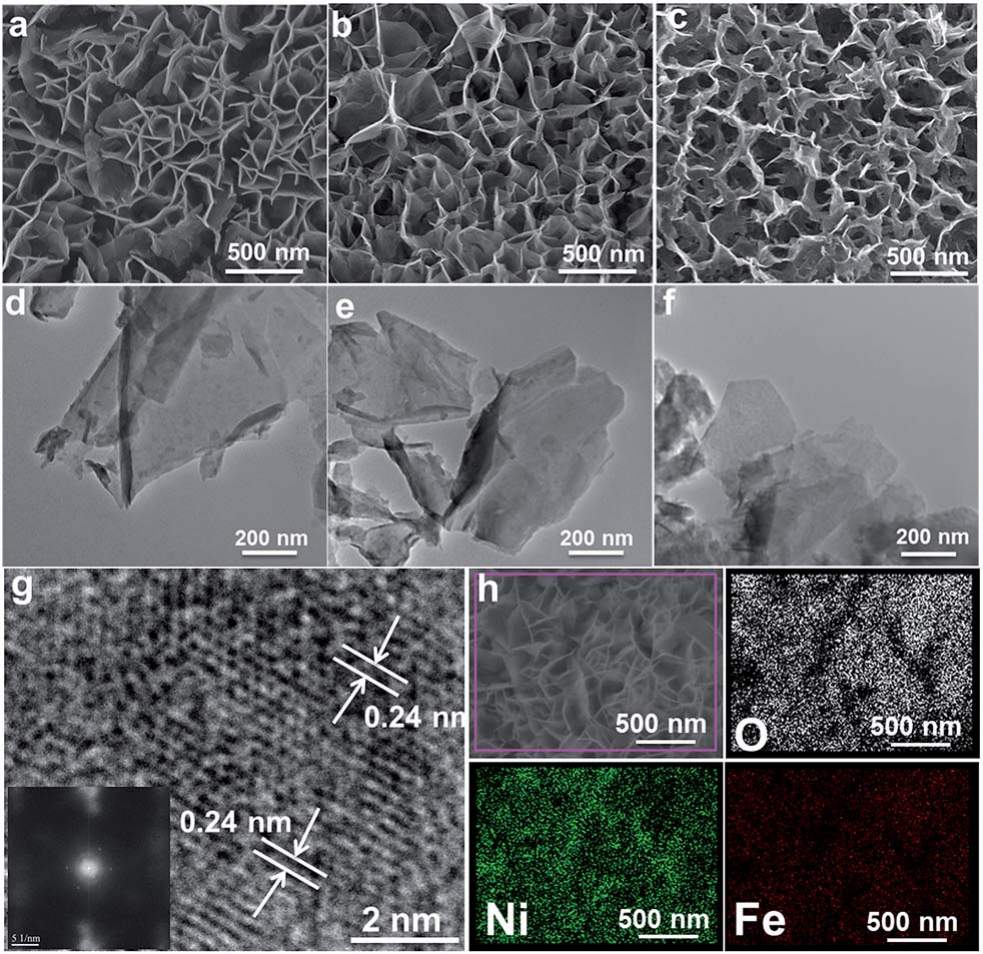
SEM and TEM images of (a and d) NiFe-LDH, (b and e) CoFe-LDH and (c and f) LiFe-LDH nanoplatelet arrays; (g) HRTEM and (h) EDS mapping images of the NiFe-LDH nanoplatelet array.

Hierarchical MFe-LDH (M = Ni, Co and Li) nanoplatelet arrays are synthesized on the surface of a foam nickel substrate *via* an electrosynthesis procedure followed by a self-oxidation process in air (Fig. 1a). The electrochemical synthesis was achieved by the following proposed reduction reaction on the working electrode: NO_3_^–^ + H_2_O + 2e^–^ → NO_2_^–^ + 2OH^–^, in which the resulting OH^–^ leads to the precipitation of M_*x*_Fe_1–*x*_(OH)_2_. The whole electrosynthesis process is finished successfully within hundreds of seconds at room temperature. The as-synthesized M_*x*_Fe_1–*x*_(OH)_2_ material is a light green color (Fig. 1b). After exposure in air for ∼1 h, the sample color changes from green to brownish, indicating the occurrence of self-oxidation of Fe^2+^ to Fe^3+^. Since it is hard to collect the MFe-LDH (M = Ni, Co and Li) powdered samples from the Ni foam to measure their XRD patterns, Fig. 1c illustrates the XRD patterns of the MFe-LDH (M = Ni, Co and Li) samples following electrosynthesis on the Ni foil substrate. The clear reflections of (003), (006) and (009) are observed for all these three MFe-LDH samples, which can be assigned to a typical LDH phase. The FT-IR technique was also used to identify the nature and symmetry of the interlayer anions of the MFe-LDHs (Fig. S1†). The spectra of all three samples show broad intense bands between 3600 and 3200 cm^–1^ due to the OH stretching mode of the hydroxyl groups in the host layers and the interlayer water molecules. The band at 1507 cm^–1^ with its accompanying band at 1359 cm^–1^ is attributed to mode ν3 of the interlayer carbonate species. Surface elemental analysis was carried out using XPS over the MFe-LDH (M = Ni, Co and Li) nanoplatelet arrays. The full XPS spectra of MFe-LDH (Fig. 1d) show peaks located at 856.0 eV (NiFe-LDH), 780.9 eV (CoFe-LDH) and 55.8 eV (LiFe-LDH), corresponding to the 2p levels of Ni^2+^ and Co^2+^ and the 1s of Li^+^, respectively. The Fe 2p_3/2_ and Fe 2p_1/2_ spin-orbital splitting photo-electrons for all three MFe-LDH samples are located at 711.0 and 724.9 eV, respectively (Fig. S2a†), indicating that the oxidation state in the MFe-LDH (M = Ni, Co and Li) nanoplatelet arrays is Fe(iii).^34^ As shown in the XPS spectra of the NiFe^2+^ hydroxide and NiFe^3+^-LDH samples (Fig. S2b†), the binding energy of Fe 2p increases from 710.0 eV to 711.0 eV, further indicating the occurrence of self-oxidation of Fe^2+^ to Fe^3+^.^49^ Based on the results above, the crystal structure of the obtained LDHs is shown in Fig. 1e. Fig. 2a–c display typical SEM images of the NiFe-LDH, CoFe-LDH and LiFe-LDH nanoplatelet arrays, respectively. Ultrathin and uniform NiFe-LDH platelets growing perpendicular to the surface of the substrate are observed, with 250–300 nm lateral length and ∼8 nm thickness (Fig. 2a). By tuning the divalent metal precursor, the CoFe-LDH (Fig. 2b) and LiFe-LDH (Fig. 2c) nanoplatelet arrays are also obtained with a similar plate-like microcrystal morphology and orientation, with a narrow particle size distribution (CoFe-LDH: ∼310 nm in lateral length and ∼8 nm in thickness; LiFe-LDH: ∼200 nm in lateral length and ∼12 nm in thickness). In addition, the low magnification SEM images (Fig. S3a, c and e†) for the MFe-LDH nanoplatelet arrays further verify the feasibility of the electrosynthesis method demonstrated here. The typical TEM images in Fig. 2d–f show individual nanoplatelets of NiFe-LDH, CoFe-LDH and LiFe-LDH, respectively. It is worth mentioning that the particle size of MFe-LDH (M = Ni, Co and Li) calculated by using the Scherrer formula (50–80 nm) is apparently smaller than that determined by SEM and TEM (200–300 nm), probably due to the presence of some amorphous LDH phase. An HRTEM image of the NiFe-LDH nanoplatelet (Fig. 2g) demonstrates that the material has a good crystallinity. An interplanar distance of 0.24 nm is measured for a single-crystal particle, corresponding to the (012) plane of the NiFe-LDH phase. The chemical compositions of as-obtained LDH nanoplatelet arrays are further determined using EDX. Fig. 2h shows the EDX mapping analysis of NiFe-LDH, from which the iron and nickel are both seen to be homogeneously distributed on the surface of sample. The EDX spectra of the MFe-LDH (M = Co, Ni and Li) nanoplatelet arrays (Fig. S3†) also display the presence of M (Ni and Co; Li cannot be defined using EDX) and Fe, which is consistent with the mapping results. In addition, the M/Fe ratios of the three samples were further studied by using an elemental analysis method (inductively coupled plasma-atomic emission spectroscopy, ICP-AES), and the obtained data are listed in Table S1.† It is found that the three samples show rather close M/Fe molar ratios: 1.91, 1.89 and 1.85 for NiFe-, CoFe- and LiFe-LDH respectively, approximately consistent with the results of the EDS analysis.

The formation process of such interesting MFe-LDH nanoplatelet arrays was further studied by controlling the reaction time. Fig. S4† shows the SEM images of nine NiFe-LDH samples obtained using different electrosynthesis durations from 5 s to 500 s. A thin layer of nanoflake-like subunits appears on the surface of the foam nickel using a short deposition time (5 s, Fig. S4a†); numerous nanoflakes are formed using a deposition time of 50 s (Fig. S4d†). As the reaction time is further prolonged, the LDH nanoflakes with well-defined plate-like morphology grow much bigger and more densely with decreasing interspace. After 300 s of electrodeposition, the whole surface of the foam nickel is covered uniformly with LDH nanoflakes (Fig. S4g†), and a much denser LDH modified surface is obtained with an even longer synthesis time (Fig. S4h-i†). Moreover, NiFe-LDH nanoplatelet arrays obtained with different electrochemical potentials (Fig. S5†) and various precursor concentrations (Fig. S6†) were studied. The optimal synthesis condition for the NiFe-LDH arrays can be determined, with a potential of –1.0 V *vs.* SCE in an electrolyte containing 0.15 M Ni(NO_3_)_2_·6H_2_O and 0.15 M Fe(SO_4_)_2_·7H_2_O, which gives a good LDH crystallinity and an ordered array morphology.

### Oxygen evolution reaction (OER)

**Fig. 3:**
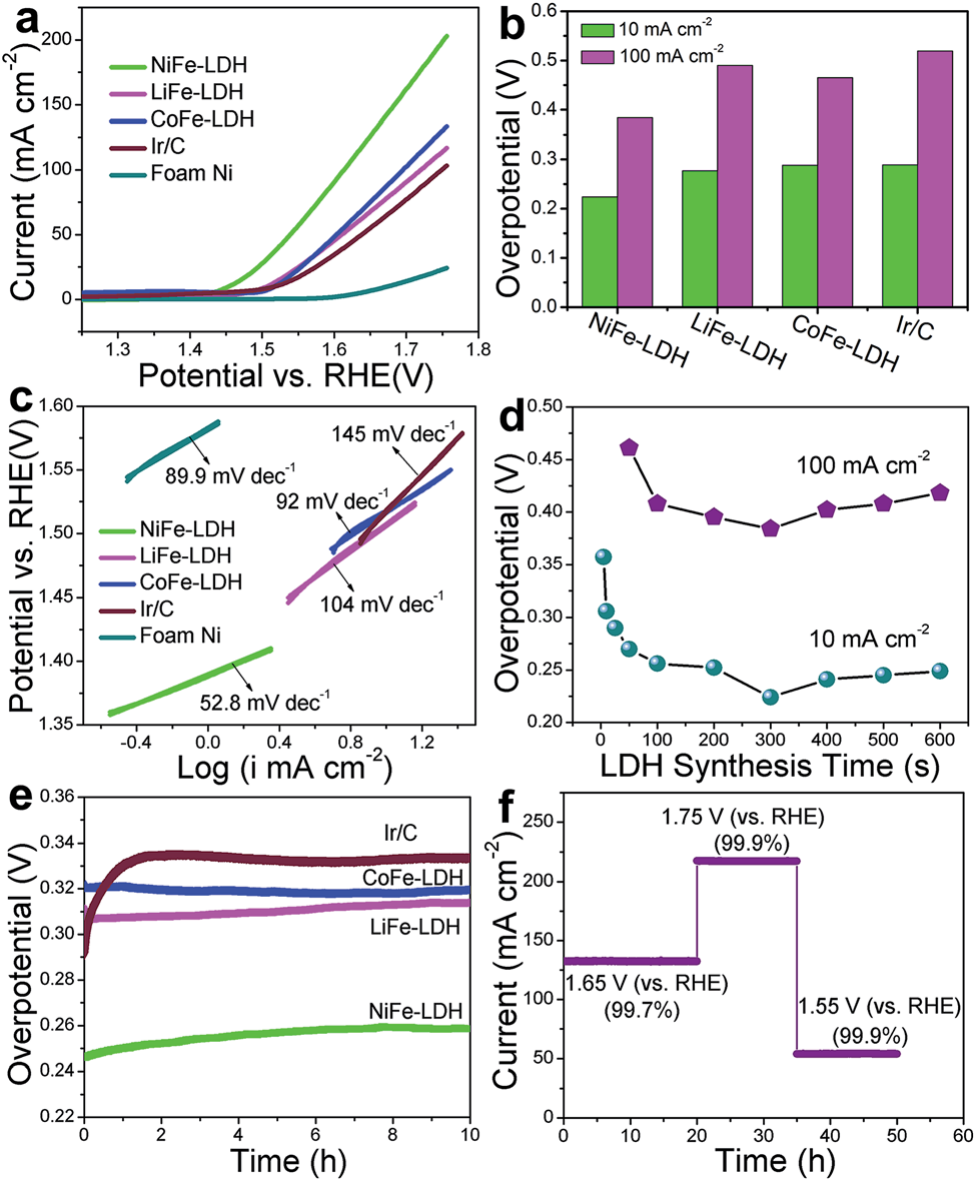
(a) Linear sweep voltammetric (LSV) curves, (b) overpotential (at 10 and 100 mA cm^–2^) and (c) Tafel plots of the MFe-LDH (M = Ni, Co and Li) nanoplatelet arrays and the commercial electrocatalyst Ir/C; (d) overpotentials (at 10 and 100 mA cm^–2^) of NiFe-LDH nanoplatelet arrays synthesized for various durations from 0 s to 600 s; (e) chronopotentiometric measurements at 10 mA cm^–2^ for the MFe-LDH (M = Ni, Co and Li) nanoplatelet arrays and commercial Ir/C for 10 h; (f) chronoamperometric curve at varied overpotentials for the NiFe-LDH nanoplatelet array.

The OER activities of the MFe-LDH (M = Ni, Co and Li) nanoplatelet arrays and the reference sample (Ir/C) in alkaline solutions were evaluated in 1 M KOH using a standard three-electrode system. The mass-loading of the Ir/C catalyst supported on the Ni foam was controlled to be the same value of the MFe-LDH (M = Ni, Co and Li) samples (1 mg cm^–2^) to give a reasonable comparison. Fig. 3a shows the polarization curves at a slow scan rate of 10 mV s^–1^ to minimize the capacitive current. The NiFe-LDH nanoplatelet array displays the lowest onset potential of the OER current and the highest current density at the same overpotential (*η*) among these four electrocatalysts, revealing the highest electrochemical activity. Overpotentials at current densities of 10 and 100 mA cm^–2^ for the various catalysts are given in Fig. 3b. At 10 mA cm^–2^, the as-prepared NiFe-LDH requires an overpotential of 224 mV, which is 64, 53, and 65 mV less than CoFe-LDH, LiFe-LDH and commercial Ir/C, respectively. Similarly, the overpotential of the NiFe-LDH sample is also the lowest even at a high current density (100 mA cm^–2^). The current density at *η* = 300 mV is 44.3 mA cm^–2^ for NiFe-LDH, which is ∼3.1, 2.6 and 3.6 times the current density for CoFe-LDH, LiFe-LDH and commercial Ir/C, respectively. The Tafel slope of the NiFe-LDH sample is 52.8 mV dec^–1^ (Fig. 3c), much smaller than that of the CoFe-LDH (92.0 mV dec^–1^), LiFe-LDH (104.0 mV dec^–1^) and Ir/C (145.0 mV dec^–1^) samples, indicating its superior OER performance. The activity of the MFe-LDHs nanoplatelet arrays was further investigated by studying their apparent turnover frequencies (TOFs) (see ESI for calculation details†). The NiFe-, CoFe-, LiFe-LDH and Ir/C samples give TOF values of 0.013, 0.0075, 0.0054 and 0.0036 s^–1^ at *η* = 300 mV (Table S2†), respectively, which implies that the NiFe-LDH nanoplatelet array has the highest activity. The actual oxygen production catalyzed by the NiFe-LDH sample at a constant current of 100 mA cm^–2^ was obtained by using the water displacement method in an air-tight reactor to measure the faradaic yield for O_2_ formation (Fig. S7†). The catalyst shows a faradaic efficiency of 99.4% after an induction period of 10 min, indicating a satisfactory energy conversion efficiency from electric energy to chemical energy. Furthermore, the OER polarization curves of MFe-LDH (M = Ni, Co and Li) electrodeposited onto a glassy carbon (GC) electrode were studied (Fig. S8†). The NiFe-LDH sample exhibits a superior OER performance, with an onset potential of 1.43 V *vs.* RHE (the same value as the Ni foam substrate) and an overpotential of 380 mV at a current density of 10 mA cm^–2^ (much lower than that of Ir/C (490 mV)). It is worth mentioning that the current density of the MFe-LDH nanoarrays supported on the Ni foam is larger than on the GC electrode at the same overpotential, which can be ascribed to the higher surface area of the Ni foam substrate.

To elucidate the intrinsic activities of the MFe-LDH (M = Ni, Co and Li) samples, density functional theory (DFT) calculations were carried out for the water oxidation reaction of these Fe-containing LDH samples (see computational details in the ESI†). Generally, it has been proposed that the electrocatalytic OER in alkaline media proceeds through multistep reactions:^50^ (1) the formation of an *OH intermediate from adsorbed H_2_O on the active sites of the catalyst; (2) a further oxidation or decomposition of *OH to *O; (3) the reaction of *O with an H_2_O molecule to produce an *OOH intermediate; (4) the release of O_2_ from *OOH. The OER performance correlates with the number of active sites and the adsorption affinity of H_2_O and intermediates. The cumulative reaction free energies (Δ*G*_298_) for the proposed reaction steps are plotted in Fig. S9.† The binding energy of the *O species (ΔG_*O_) is larger than the other species for all these three MFe-LDHs, indicating that the oxidation of *O to *OOH is the rate-determining step. The ΔG_*O_ value increases in the following sequence: NiFe-LDH (1.506 eV) < CoFe-LDH (1.577 eV) < LiFe-LDH (1.653 eV). This yields overpotentials of 0.276 V, 0.347 V and 0.423 V for NiFe-, CoFe-, and LiFe-LDH, respectively, which is consistent with the order of the experimental values. The results show that the NiFe-LDH sample gives the most thermodynamically favored reaction pathway (the lowest minimum overpotential), accounting for the best electrochemical performance in OER catalysis.

The OER performance of NiFe-LDH nanoplatelet arrays synthesized with different times was also studied. The corresponding LSV curves and Tafel plots are shown in Fig. S10.† The overpotential of NiFe-LDH firstly decreases gradually along with the increase in LDH deposition time and reaches a minimum for the NiFe-LDH (300 s) sample (Fig. 3d). However, the measured overpotential both at 10 and 100 mA cm^–2^ increases significantly with further elongation of the deposition time. This result demonstrates that the coating of LDH at a suitable level can effectively enhance its OER activity, while an excess of LDH incorporation leads to a decreased catalytic efficiency, which may result from a decrease in active site exposure and charge transfer (Fig. S4h-i†). To further understand this trend, the electrochemical double layer capacitance was measured to determine the electrochemical surface area (ECSA) of NiFe-LDH samples with different mass loadings (the linear slope of capacitive current *vs.* scan rate is equivalent to twice the double layer capacitance *C*_dl_^35^) (Fig. S11†). It is found that the *C*_dl_ of NiFe-LDH firstly increases gradually along with the increase in LDH deposition time and reaches a maximum for the NiFe-LDH (300 s) sample. However, the *C*_dl_ decreases significantly with further elongation of the deposition time. The results indicate that an excess of LDH loading leads to a decreased electrochemical surface area and a resulting decrease in catalytic efficiency. Moreover, the electrochemical impedance spectroscopy (EIS) spectra for the NiFe-LDH samples provide additional information about the charge transport properties (Fig. S12†). The resistance increases slowly within the first 300 s, but increases rapidly from 300 s to 500 s, indicating a decreased charge transport with a dense loading of LDH. Therefore, the LDH nanoplatelet arrays grown on Ni foam with a suitable level would effectively enhance the OER activity, while an excess of LDH loading leads to a decreased electrochemical surface area (ECSA) and a decrease in charge transport. A durability test of the MFe-LDH nanoplatelet arrays was carried out by means of a chronopotentiometry measurement at 10 mA cm^–2^ (Fig. 3e). The operating overpotential for the MFe-LDH (M = Ni, Co and Li) nanoplatelet arrays is nearly constant and only increases by 2–5% after 10 h of testing, indicating a good durability of the MFe-LDH samples in alkaline solution. In contrast, the overpotential of the state-of-the-art OER catalyst Ir/C increases from 285 to 334 mV in only 2 h. Fig. 3f shows the current density curves as a function of time recorded at varied potentials over 50 h using the NiFe-LDH electrode. It is found that the current density of the OER remains constant at each given potential (<1% current decay), further demonstrating the significantly long-term stability of the NiFe-LDH electrocatalyst.

The OER performances of the MFe-LDHs synthesized by an electrochemical method in this work are compared with other previously reported LDH-based electrocatalysts (Table 1), and show obvious advantages. Firstly, previous LDHs-based electrodes normally require multi-step, time- and energy-consuming procedures, while the electro-synthesized MFe-LDHs can be obtained at room temperature within hundreds of seconds (<300 s). Secondly, the MFe-LDH nanoplatelet arrays with hierarchical architectures in this work guarantee sufficient exposure of the active sites and facilitate a fast mass/charge transport, accounting for the largely-enhanced OER behavior. The onset potential and overpotential at 10 mA cm^–2^ of the NiFe-LDH nanoplatelet array in this work are superior to those of previously reported NiFe-LDH based catalysts, such as pure LDH particles,^45^ and exfoliated NiFe-LDH nanosheets^37^ as well as LDHs-based nanocomposites.^39^–^43^ In addition, this electrosynthesized NiFe-LDH electrode is a carbon free system compared with most reported OER catalysts.

**Table 1:** Comparison of the synthesis methods of Fe-containing LDHs and their OER activity in alkaline medium

Catalyst	Method (synthesis time)	Onset potential (V *vs.* RHE)	*η* at *J* = 10 mA cm^–2^ (mV)	*J* at *η* = 300 mV (mA cm^–2^)	Stability	Reference
NiFe-LDH array	*In situ* growth (>24 h)	∼1.46	∼230	∼40	10 h at 1.5 V (∼97%)	38
Exfoliated NiFe-LDH nanosheet	Hydrothermal (>24 h)	∼1.53	∼290	∼9	12 h at 10 mA cm^–2^ (∼95%)	37
NiFe-LDH particle	Hydrothermal (>24 h)	∼1.43	∼260	—	—	45
NiFe-LDH array	Electrosynthesis + aging (>24 h)	∼1.46	—	—	12 h at 10 mA cm^–2^ (∼99%)	48
NiFe-LDH/graphene particle	Hydrothermal (>24 h)	∼1.44	∼205	—	1.5 h at 5 mA cm^–2^ (∼99%)	39
NiFe-LDH/graphene Ni foam	*In situ* growth (>24 h)	1.47	325	44	2.5 h at 10 mA cm^–2^ (∼99%)	40
nNiFe LDH/NGF	Hydrothermal (>24 h)	—	337	45	10 h at 1.58 V (∼99%)	41
Ni_2/3_Fe_1/3_-rGO	Hydrothermal (>24 h)	—	∼210	40	10 h at 10 mA cm^–2^ (∼97%)	42
NiFe-LDH/CQD particle	Hydrothermal (>24 h)	∼1.46	235	∼35	0.83 h at 2.5 mA cm^–2^ (∼97%)	43
NiFe-LDH/CNT particle	Hydrothermal (>24 h)	∼1.45	∼240	∼45	0.28 h at 2.5 mA cm^–2^ (∼100%)	36
NiFe-LDH array	Electro-synthesis (<300 s)	∼1.43	224	44	50 h at 1.55–1.75 V (∼100%)	This work

### Electrochemical oxidation of hydrazine, methanol and ethanol

**Fig. 4:**
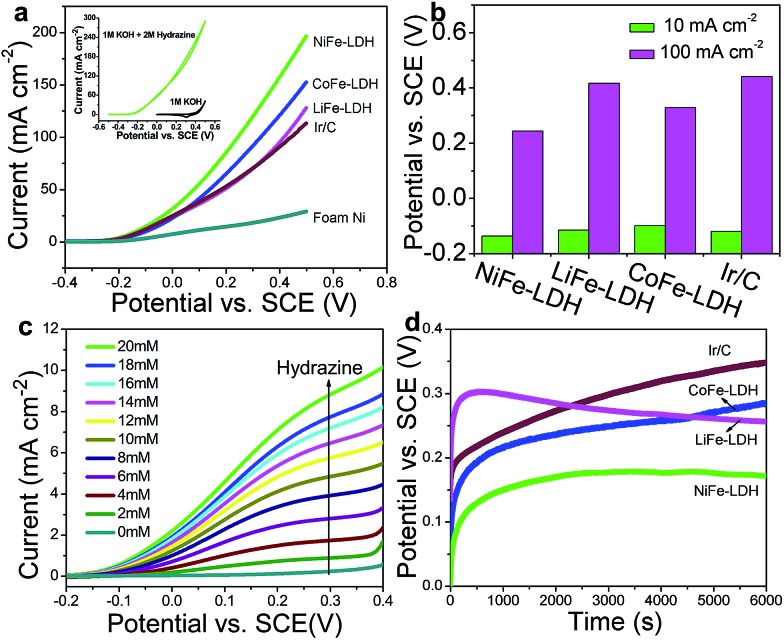
(a) LSV curves of the MFe-LDH (M = Ni, Co and Li) nanoplatelet arrays, Ir/C and foam nickel substrate in 1 M KOH with 2 M hydrazine (inset: CV curves of NiFe-LDH in 1 M KOH and 1 M KOH with 2 M hydrazine); scan rate: 0.1 V s^–1^. (b) The required potentials of different electrocatalysts to reach current densities of 10 and 100 mA cm^–2^. (c) LSV curves of the NiFe-LDH electrode in 1 M KOH solution with various concentrations of hydrazine; scan rate: 0.1 V s^–1^. (d) Chronopotentiometric measurements at 10 mA cm^–2^ over MFe-LDH (M = Ni, Co and Li) nanoplatelet arrays and commercial Ir/C.

To validate the universal electrocatalytic activity of the MFe-LDH nanoplatelet arrays, their catalytic performances toward electro-oxidation reactions of other small molecules were subsequently evaluated. The exploration of efficient catalysts for the electro-oxidation of hydrazine is appealing because of its large hydrogen density delivery, high theoretical cell voltage and absence of CO_2_ emission.^51^,^52^ CV and LSV curves of the MFe-LDH (M = Ni, Co and Li) nanoplatelet arrays, Ir/C and foam nickel substrate in 1 M KOH with 2 M hydrazine were measured. The oxidation current was normalized to the geometric surface areas; this allows the current density to be directly used to compare the catalytic activity of the different samples. The CV curves of the NiFe-LDH nanoplatelet arrays (Fig. 4a, inset) show a significant negative-shift and anodic current increase with the addition of hydrazine, highlighting the remarkable electrocatalytic activity (onset potential ∼0.2 V *vs.* saturated calomel electrode, SCE). Furthermore, the LSV curves (Fig. 4a) of the different MFe-LDH arrays and Ir/C catalyst in 1 M KOH solution with 2 M hydrazine demonstrate that their electrocatalytic abilities are in the following sequence: NiFe-LDH > CoFe-LDH > LiFe-LDH ≥ Ir/C, which is the same order as for the OER performances. The potentials at current densities of 10 and 100 mA cm^–2^ for the different catalysts are given in Fig. 4b. In the case of 100 mA cm^–2^, the NiFe-LDH sample requires a potential of 244 mV, which is 85, 173, and 198 mV less than the CoFe-LDH, LiFe-LDH and commercial Ir/C samples, respectively. It is worth mentioning that even with a low hydrazine concentration, a linear relationship between the peak current over NiFe-LDH and the hydrazine concentration in the range 2–20 mM is observed (Fig. 4c and Fig. S13†), further demonstrating its remarkable catalytic activity.

To evaluate the electrocatalytic activity and stability of MFe-LDH under continuous operating conditions, long-term chronoamperometric tests at 10 mA cm^–2^ were carried out in a 1.0 M KOH + 2.0 M hydrazine solution (Fig. 4d). It is found that the required potential of the NiFe-LDH sample at the same current density is smaller than those of the CoFe-LDH, LiFe-LDH and Ir/C samples, highlighting a significantly improved electrocatalytic activity. Furthermore, the NiFe-LDH nanoplatelet array catalyst exhibits a largely enhanced long-term durability for hydrazine electro-oxidation. The electrochemical behavior of the MFe-LDH nanoplatelet arrays was further verified by the electrochemical oxidation of methanol and ethanol, which is estimable for low temperature direct fuel cell reactions. As shown in Fig. S14,† the oxidation current is largely enhanced for the NiFe-LDH electrode in the presence of either methanol or ethanol. In addition, the sequence of electro-oxidation onset potentials for the small molecules studied here is as follows: hydrazine > water > methanol > ethanol. All the onset potentials for these molecules are relatively low compared with previously reported values,^6^,^11^,^41^ demonstrating the high activity of NiFe-LDH nanoarrays toward small molecule oxidations. This indicates that NiFe-LDH nanoarrays can be used as highly efficient electro-oxidation catalysts for energy conversion devices such as metal–air batteries and fuel cells.

## Discussion

**Fig. 5:**
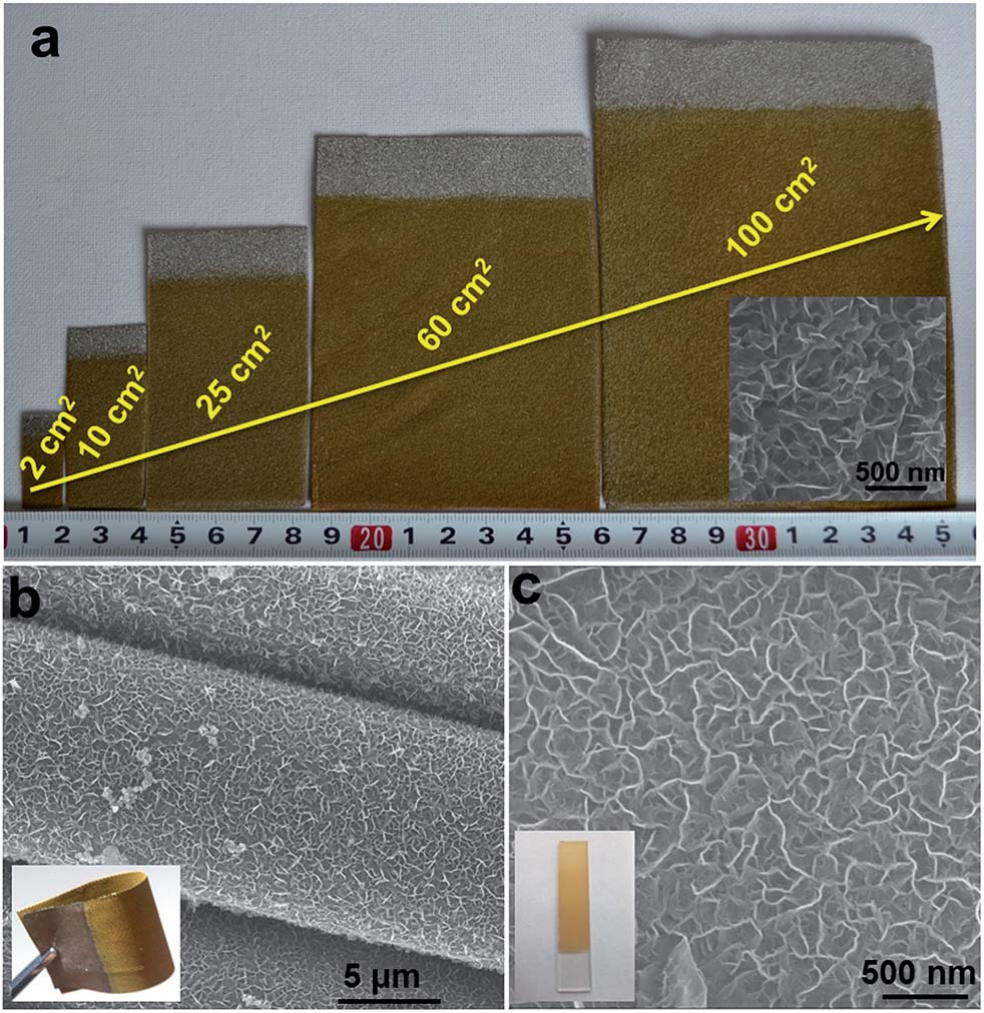
(a) Photographs of NiFe-LDH nanoplatelet arrays synthesized on foam nickel substrates at various scales (inset: the SEM image of the NiFe-LDH sample on the 100 cm^2^ substrate); synthesis time for all the samples is 300 s at room temperature. SEM images for the NiFe-LDH nanoplatelet arrays on (b) a conducting cloth and (c) FTO substrate (inset: their corresponding photographs).

Although various LDH-based materials have been investigated as OER catalysts and exhibited promising electrochemical properties, the majority of LDH-based catalysts so far are difficult to be applied in practical applications due to their complicated preparation processing, high cost and uncontrollable nanostructures (*e.g.*, particles have been mostly investigated, but suffer from aggregation). In this work, the hierarchical NiFe-LDH material demonstrates a promising performance towards small molecule oxidations. This can be attributed to the ordered nanoplatelet array structure, which facilitates electrolyte diffusion and electron transport. This is hardly achieved by random particles deposited on electrodes, as reported previously. Furthermore, the good chemical stability of LDHs in basic environments and the good combination with the substrate greatly enhance the cycling stability of the NiFe-LDH catalyst. As shown in Fig. S15,† after 20 h and 5 h continuous OER and hydrazine oxidation testing, respectively, the surface of the NiFe-LDH electrode maintains its original hierarchical array architecture. The high electro-oxidation catalytic activity along with the excellent cycling stability of the MFe-LDHs meets the requirements of both high efficiency and long endurance simultaneously, which are prerequisites for practical applications. In addition, as shown in Fig. 5a, NiFe-LDHs nanoplatelet arrays directly growing on the foam nickel substrate can be effectively scaled up from 2 cm^2^ to 100 cm^2^ with a uniform and homogeneous surface morphology. This electrosynthesis method is also adequate for the fabrication of NiFe-LDH nanoplatelet arrays on other conducting substrates, such as conducting clothes and glasses (Fig. 5b and c). The OER performances of NiFe-LDH arrays grown on a Ni foam, a conducting cloth and FTO were also studied. It is found that the NiFe-LDH/Ni foam displays the highest current density at the same overpotential (*η*) (Fig. S16†), which can be ascribed to the large specific surface area and good conductivity of the Ni foam. Therefore, the electrochemical preparation of MFe-LDH array electrodes has the advantages of simplicity, fast operation, low cost and high yield, and can serve as a general strategy for the scalable manufacture of electrode materials. Besides Fe-containing LDHs, this electrosynthesis method can be further extended to the preparation of LDH nanoplatelet arrays with other compositions (*e.g.*, Mn-containing LDHs, Co-containing LDHs) on the surface of various conducting substrates, which will be demonstrated in our upcoming future work.

## Conclusion

In summary, hierarchical MFe-LDHs (M = Ni, Co and Li) nanoplatelet arrays have been successfully obtained *via* a fast and effective electrosynthesis method, which allows the crystallization of the target materials in one synthetic step at room temperature. The obtained NiFe-LDH array displays excellent catalytic activity and robust durability for small molecule electro-oxidations (H_2_O and N_2_H_4_), superior to most reported transition metal oxides/hydroxides catalysts as well as noble metal catalysts such as Ir/C (20 wt%). Considering the catalytic capability toward the oxidation reaction of small fuel molecules, Fe-containing LDH electrodes fabricated by this electrosynthesis method are promising for direct use in water-splitting devices and fuel cells. Moreover, we believe that the reported synthetic approach can be further extended to other types of LDH-based nanostructures for advanced performances in the fields of energy conversion and storage.

## Supplementary Material

Supplementary information
