# Supplementary material for: Fast electrosynthesis of Fe-containing layered double hydroxide arrays toward highly efficient electrocatalytic oxidation reactions
Source: Chem Sci. 2015 Aug 12;6(11):6624–31. doi: 10.1039/c5sc02417j (PMC5802274; doi:10.1039/c5sc02417j)
Supplement: Supplementary file 1 [file SC-006-C5SC02417J-s001.pdf]

Electronic Supplementary Material (ESI) for Chemical Science.
This journal is © The Royal Society of Chemistry 2015

Electronic Supplementary Material (ESI) for Chemical Science.

This journal is © The Royal Society of Chemistry 2015

Supporting Information for

Fast Electrosynthesis of Fe-Containing Layered Double Hydroxides Arrays toward Highly Efficient Electrocatalytic Oxidation Reactions †

Zhenhua Li,[a] Mingfei Shao,*[a] Hongli An,[a] Zixuan Wang,[a] Simin Xu,[a] Min Wei,*[a] David G. Evans[a] and Xue Duan[a]

[a.] State Key Laboratory of Chemical Resource Engineering, Beijing University of Chemical Technology, Beijing 100029, China

*Corresponding author. Fax: 86-10-64425358; Tel: 86-10-64412131.

E-mail: shaomf@mail.buct.edu.cn (M. Shao); weimin@mail.buct.edu.cn (M. Wei)

Contents

1. Experimental Section S2
   1.1 Materials S2
   1.2 Preparation of MFe-LDH (M=Ni, Co and Li) nanoplatelet arrays S2
   1.3 Characterization S2
   1.4 Electrochemical performance measurements S3
   1.5 Computational details S4
   1.6 QCM Measurement S5
   1.7 TOF calculation S5
   1.8 Tafel calculation and Onset potential S5
2. Figures S6
3. References S15

1. Experimental Section

1.1 Materials

$Ni(NO_3)_2{\cdot}6H_2O$, $Co(NO_3)_2{\cdot}6H_2O$, $LiNO_3$, $Fe(SO_4)_2{\cdot}7H_2O$, KOH and 20 wt% Ir/C catalyst were purchased from Aldrich Ltd. (Shanghai, China). Dehydrated ethanol, methanol and hydrazine hydrate were purchased from Beijing Chemical Corp. The water used in all experiments was purified through a Millipore system.

1.2 Preparation of MFe-LDH (M=Ni, Co and Li) nanoplatelet arrays

The MFe-LDH (M= Ni, Co and Li) nanoplatelet arrays was prepared using a facile electrosynthesis method. Typically, nickel foam (10 mm × 40 mm × 1 mm) was pretreated with 2 M HCl solution, absolute ethanol, acetone and deionized water (each for 15 min) to ensure a clean surface. The Ni foam was used as the working electrode and placed in an electrochemical cell in a three-electrode configuration, by using Pt wire and saturated calomel electrode (SCE) as the counter and reference electrode, respectively. The electrolyte for the electrosynthesis of NiFe-LDH was obtained by dissolving $Ni(NO_3)_2{\cdot}6H_2O$ (0.15 M) and $Fe(SO_4)_2{\cdot}7H_2O$ (0.15 M) in 50 mL of distilled water which was agitated by a magnetic stirrer at 500 r/min with a continuous N_2 flow to prevent the oxidation of Fe^{2+}. The potentiostatic deposition was carried out at a potential of −1.0 V *vs*. SCE. The resulting NiFe-LDH nanoplatelet array was withdrawn and rinsed thoroughly with distilled water. The CoFe-LDH and LiFe-LDH nanoplatelet arrays were prepared *via* the similar method, by replacing $Ni(NO_3)_2{\cdot}6H_2O$ (0.15 M) with $Co(NO_3)_2{\cdot}6H_2O$ (0.15 M) or $LiNO_3$ (0.15 M), respectively. The total mass of MFe-LDH grown on Ni foam was determined by using an analytical balance. In this work, MFe-LDH (M=Ni, Co and Li) were also electrodeposited onto glassy carbon (GC) electrode with the same method, and the mass was determined by using quartz crystal microbalance (QCM), as shown in Fig. S17. As a reference, 5 μL of the Ir/C (20 wt%

of Ir) solution was drop-casted onto the surface of GC electrode and dried in air. The loaded Ir onto GC electrode was determined to be ~160 μg cm^{-2}.

The Ir/C electrode was used as a reference sample, which was prepared by a dip-coating method.[1,2] Typically, 0.1 g of 20 wt% Ir/C catalyst was dispersed in 2 mL of ethanol, followed by addition of 0.2 g of 5 wt% nafion solution. The mixture was treated by ultrasonic for 30 min. Subsequently, a piece of clean nickel foam (1 cm × 1 cm × 0.1 cm) was immersed into the above solution for 10 min, withdrawn and dried by a nitrogen gas flow. The mass-loading of Ir/C catalyst was controlled by repeating the above deposition process for several cycles to obtain the same value of LDH sample (1 mg cm^{-2}).

1.3 Characterization

X-ray diffraction patterns of the MFe-LDH (M= Ni, Co and Li) nanoplatelet arrays were collected on a Shimadzu XRD-6000 diffractometer using a Cu Kα source, with a scan step of 0.02° and a scan range between 3° and 70°. FTIR spectra were recorded on a NICOLET NEXUS470 Fourier transform infrared spectrometer. X-ray photoelectron spectra (XPS) were performed on a Thermo VG ESCALAB 250 X-ray photoelectron spectrometer at a pressure of about 2×10^{-9} Pa using Al Kα X-rays as the excitation source. The morphology of MFe-LDH nanoplatelet arrays was investigated using a scanning electron microscope (SEM; Zeiss SUPRA 55) with an accelerating voltage of 20 kV, combined with energy dispersive X-ray spectroscopy (EDX) for the determination of metal composition. Transmission electron microscopy (TEM) images were recorded with Philips Tecnai 20 and JEOL JEM-2010 high-resolution transmission electron microscopes. The accelerating voltage was 200 kV in each case.

1.4 Electrochemical performance measurements

All the electrochemical experiments were carried out on a CHI660E electrochemical workstation (Shanghai Chenhua Instrument Co., China) in a three-electrode electrochemical cell using 1 M KOH aqueous solution as electrolyte at room temperature. The MFe-LDH (M=

Ni, Co and Li) nanoplatelet arrays on foam nickel was used as the working electrode. A Pt wire and an Hg/HgO electrode were used as the counter and reference electrode, respectively. The ECSA values of NiFe-LDH samples with different mass loading were measured. The CV potential window ranges in 0.9−1.0 V *vs.* RHE (1 M KOH), and the scan rates are 20, 40, 60, 80, and 100 mV s^{-1}, respectively. The double layer capacitance (C_{dl}) is estimated by plotting the ΔJ (J_a–J_c) at 0.95 V against the scan rate, where J_a and J_c are the anodic and cathodic current density respectively, and the slope is twice of C_{dl}.

1.5 Computational details

Model construction: The bulk LiFe-LDH, CoFe-LDH and NiFe-LDH are built with the space group of $p\bar{3}m1$. The lattice parameters are then set as follows: $\alpha = \beta = 90°$, $\gamma = 120°$; the other three lattice parameters are set according to the powder X-ray diffraction observations. The supercells of LiFe-LDH, CoFe-LDH and NiFe-LDH are 6 × 3 × 1 in the *a*-, *b*- and *c*-directions, respectively. Carbonate anions are put into the interlayer space of LiFe-LDH, CoFe-LDH and NiFe-LDH to keep the model neutral. Then, after the geometries of these three LDHs are optimized, the (003) surfaces of LiFe-LDH, CoFe-LDH and NiFe-LDH are cleaved, containing one layer of LDHs matrices and one layer of carbonates. A vacuum layer of 15 Å is added in each model. Then, the radical O, OH, and OOH are put onto the LDHs surface, being the model of *O, *OH and *OOH.

Computational details: All the calculations are performed with the CASTEP code in the Materials Studio version 6.1 software package (Accelrys software inc.: San Diego, CA). The density functional theory (DFT) calculations are performed using a plane wave implementation at the generalized gradient approximation (GGA) Perdew-Burke-Ernzerhof (PBE) level. The structure optimization is based on the following points: an energy tolerance of 10^{-5} eV per atom; a maximum force tolerance of 0.03 eV/Å; a maximum displacement

tolerance of 10^{-3} Å. The density of states of each intermediate of LiFe-LDH, CoFe-LDH and NiFe-LDH, are calculated.

1.6 QCM Measurement

Quartz crystal microbalance measurements were performed as follows. A gold-coated quartz crystal with the geometrical area of 0.5 cm^2 was used as the substrate with platinum wire and SCE as respective counter and reference electrodes. The MFe-LDH (M= Ni, Co and Li) were electrodeposited on the substrate with an optimized deposition time of 300s. The change in mass per unit area (Δm) was calculated from the changes in resonance frequency (Δf), using the Sauerbrey equation: $\Delta f = -2 f_0^2 \Delta m / [A\ (\mu\rho)^{0.5}]$,

where f_0 is the resonant frequency of the quartz resonator, A is the area of the gold coated onto the crystal, μ is the shear modulus of the quartz and ρ is density of the quartz.

1.7 TOF calculation

The TOF value of the LDH samples was calculated based on the following equation:

$$TOF = \frac{J \times A}{4 \times F \times n} \quad (1)$$

where J is the current density at an overpotential of 300 mV in mA cm^{-2}; A is the area of LDHs electrode; F is the faraday constant; n is molar number of the active material deposited onto the GC electrode.

The mass activity (MA) is calculated from the equation:

$$MA = \frac{J}{m} \quad (2)$$

where J is the current density at an overpotential of 300 mV in mA cm^{-2}; m is the mass of active material deposited onto the GC electrode.

1.8 Tafel calculation and Onset potential

The Tafel curves were plotted from the corresponding OER polarization curves,[3] according to the Tafel equation: $\eta = b \log (j/j_0)$, where η is the potential; b is the Tafel slope; j

is the current density, and j_0 is the exchange current density. The onset potential of NiFe-LDH nanoplatelet arrays was obtained from the polarization curve, at the point where the corresponding current rises sharply, indicating the start of water oxidation.[4,5]

2. Figures

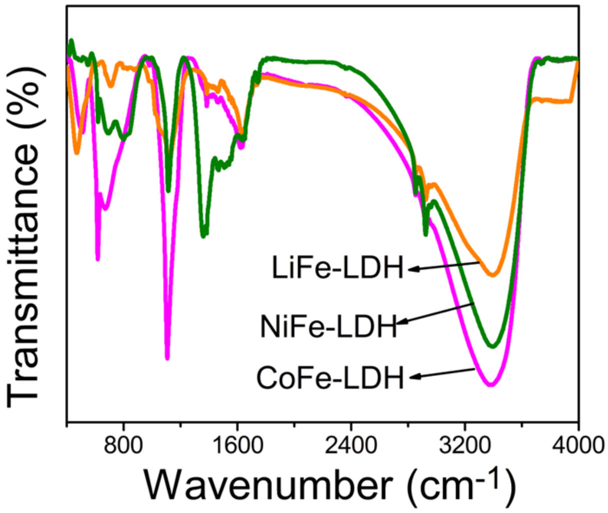

Fig. S1 FT-IR spectra of the MFe-LDHs (M= Ni, Co and Li).

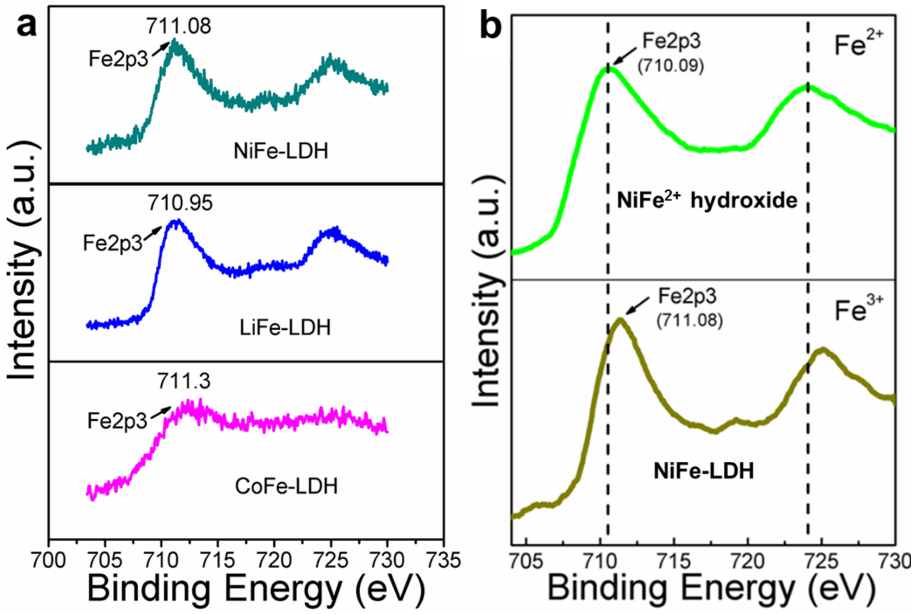

Fig. S2 a) The Fe 2$p_{3/2}$ and Fe 2$p_{1/2}$ spin-orbital splitting photo-electrons for the MFe-LDHs (M= Ni, Co and Li). b) Fe 2p XPS spectra for NiFe^{2+} hydroxide and NiFe-LDH.

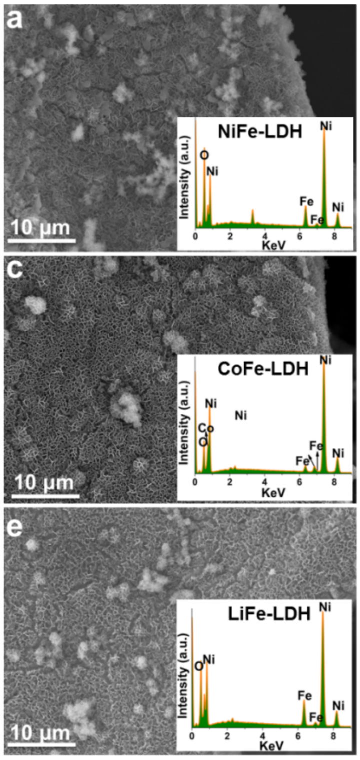

b

Element	Weight ratio (%)	Atom ratio (%)
O K	36.99	67.95
K K	0.41	0.30
Fe K	16.28	8.57
Ni K	46.32	23.19

d

Element	Weight ratio (%)	Atom ratio (%)
C K	6.55	15.20
O K	31.81	55.43
Fe K	4.69	2.34
Co K	9.47	4.48
Ni K	47.48	22.55

f

Element	Weight ratio (%)	Atom ratio (%)
O K	50.05	78.23
Fe K	22.49	10.07
Ni K	27.46	11.70

Fig. S3 The EDX spectra and corresponding elemental contents of (a, b) NiFe-LDH, (c, d) CoFe-LDH, (e, f) LiFe-LDH nanoplatelet arrays.

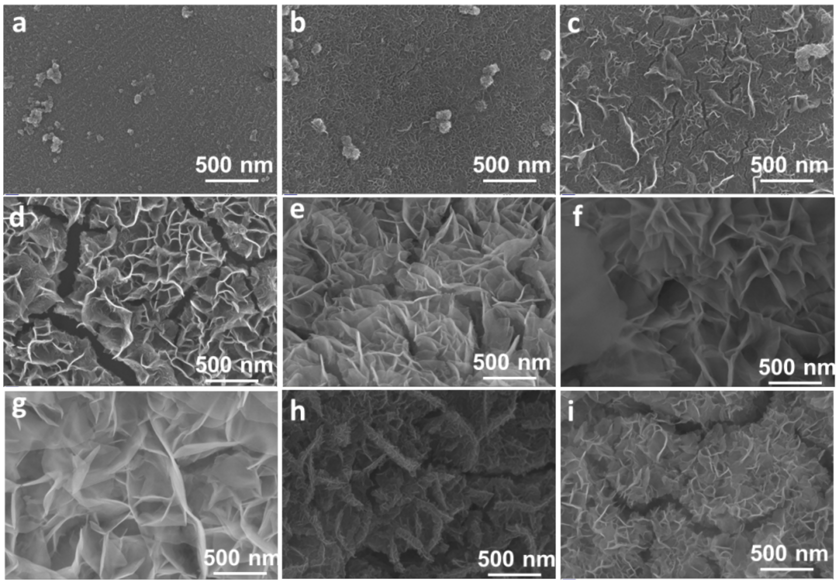

Fig. S4 SEM images of NiFe-LDH samples obtained with electrosynthesis duration from 5 s to 500 s: (a) 5 s, (b) 10 s, (c) 25 s, (d) 50 s, (e) 100 s, (f) 200 s, (g) 300 s, (h) 400 s, (i) 500 s.

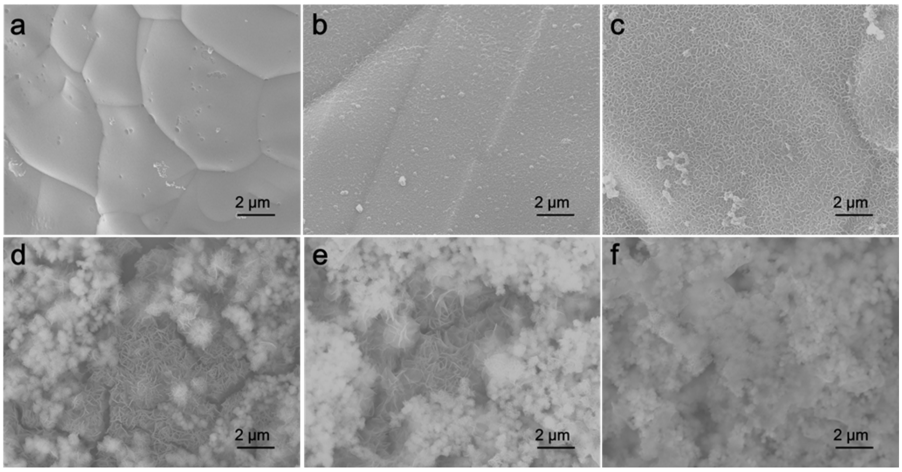

Fig. S5 SEM images of NiFe-LDH samples obtained with different electrochemical potential: (a) -0.1 V, (b) -0.5 V, (c) -0.75 V, (d) -1.5 V, (e) -2 V and (f) -3 V with the electrodeposition duration for 300 s.

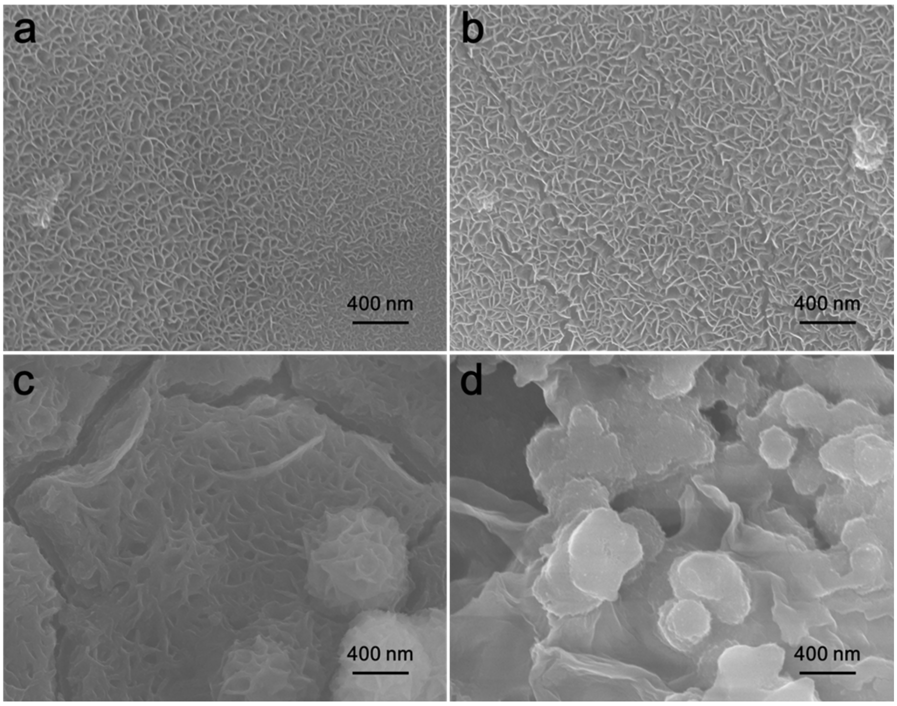

Fig. S6 SEM images of NiFe-LDH samples obtained with different concentrations of precursor: (a) 0.015 M, (b) 0.075 M, (c) 0.375 M and (d) 0.75 M for $Ni(NO_3)_2 \cdot 6H_2O$ and $Fe(SO_4)_2 \cdot 7H_2O$ with the electrodeposition duration for 300 s.

Table S1. M/Fe molar ratio for the MFe-LDH (M= Ni, Co and Li) nanoplatlet arrays.

MFe-LDH	M/Fe molar ratio
NiFe-LDH	1.91
CoFe-LDH	1.89
LiFe-LDH	1.85

Table S2. The turnover frequency values (TOF) and mass activity of MFe-LDHs nanoplatelet arrays

	NiFe-LDH	CoFe-LDH	LiFe-LDH	Ir/C
Current density (*J*) at η=300 mV (mA cm^{-2})	4.3	2.9	2.1	1.2
Mass (μg cm^{-2})	161.6	163.2	129.3	160 (Ir)
Mass activity	26.6	17.76	16.24	7.5
TOF (s^{-1})	0.013	0.0075	0.0054	0.0036

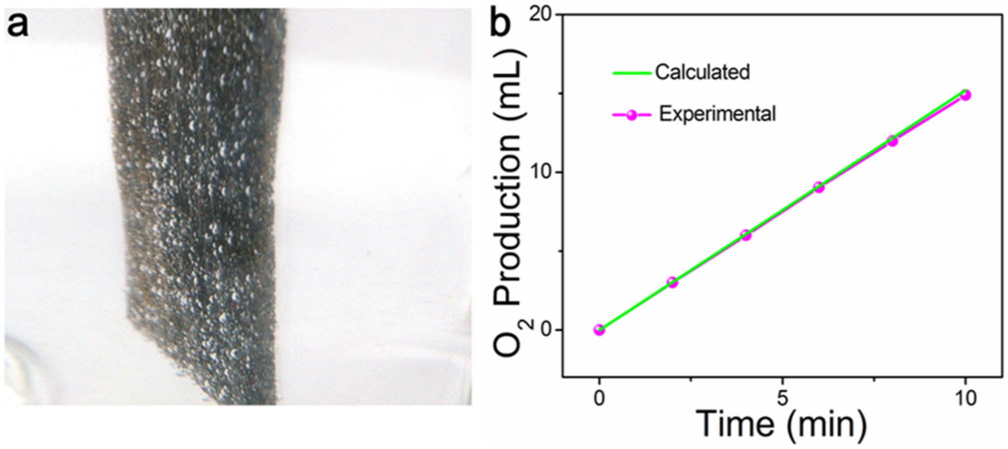

Fig. S7 (a) A typical photo of O_2 production from the surface of NiFe-LDH/Ni foam. (b) Calculated and actual oxygen production catalyzed by NiFe-LDH at a constant current of 100 mA cm^{-2}.

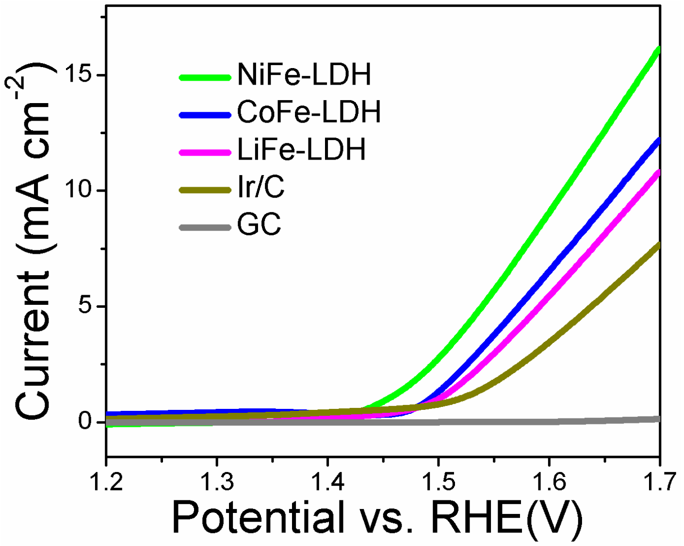

Fig. S8 Linear sweep voltammetric (LSV) curves for MFe-LDH (M= Ni, Co and Li) nanoplatelet arrays and commercial Ir/C deposited on the GC electrodes and blank GC electrde in 1 M KOH solution.

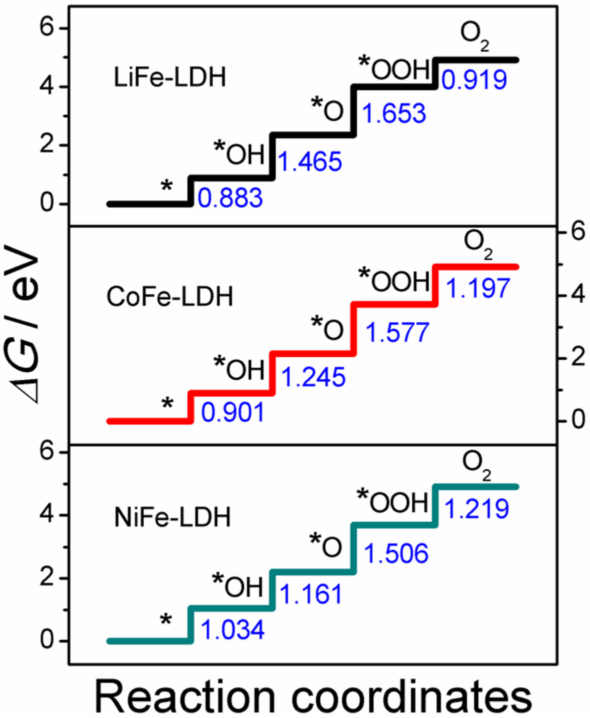

Fig. S9 Standard free energy diagrams for the oxygen evolution reaction over NiFe-LDH, CoFe-LDH and LiFe-LDH, respectively.

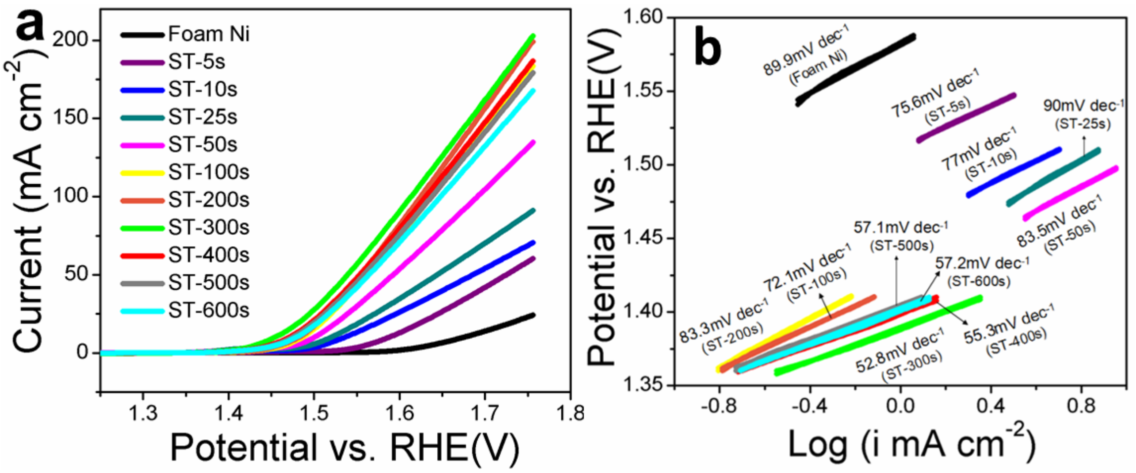

Fig. S10 (a) Linear sweep voltammetric and (b) Tafel plots of NiFe-LDH nanoplatelet arrays synthesized with various duration from 0 s to 600 s.

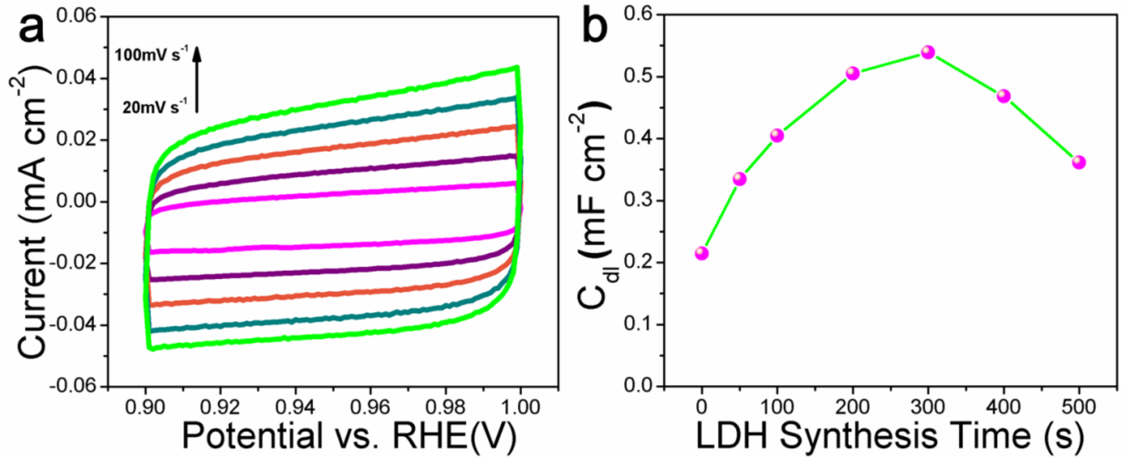

Fig. S11 (a) CV curves at various scan rates in the potential range 0.9–1.0 V *vs*. RHE for NiFe-LDH (300 s). (b) The double layer capacitance C_{dl} for NiFe-LDH samples synthesized with different electrodeposition durations.

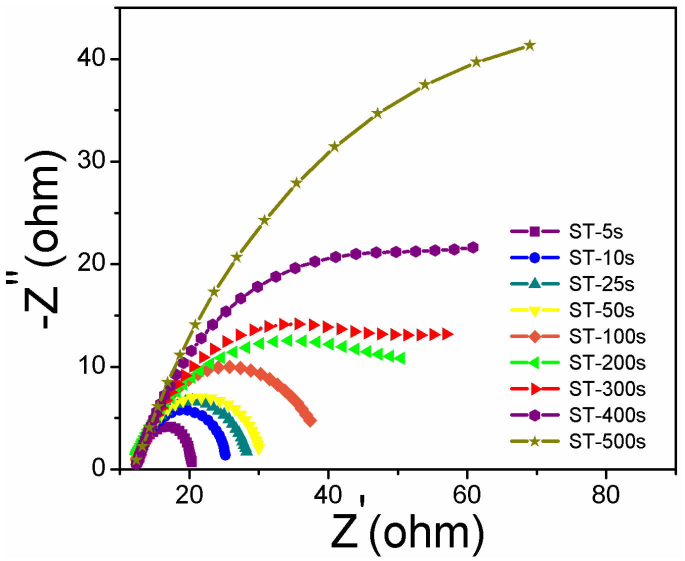

Fig. S12 Electrochemical impedance spectroscopy (EIS) spectra for NiFe-LDH with different synthesis time from 5 s to 500 s.

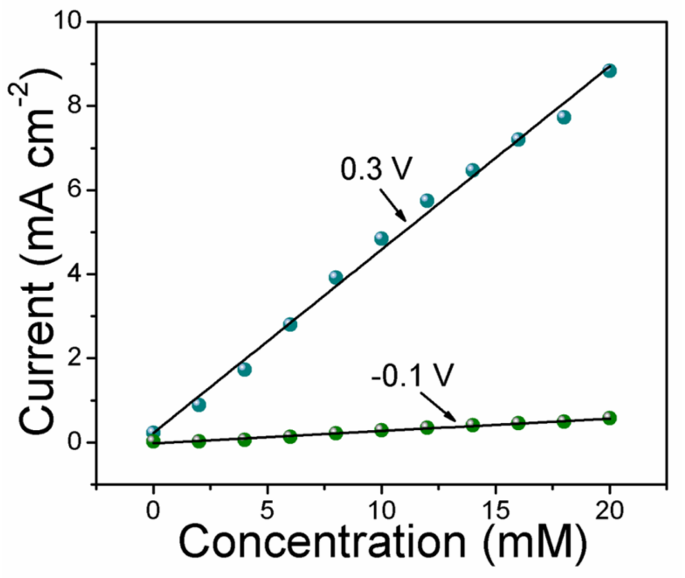

Fig. S13 Plots of current density *vs.* hydrazine concentration at −0.1 V and 0.3 V, respectively.

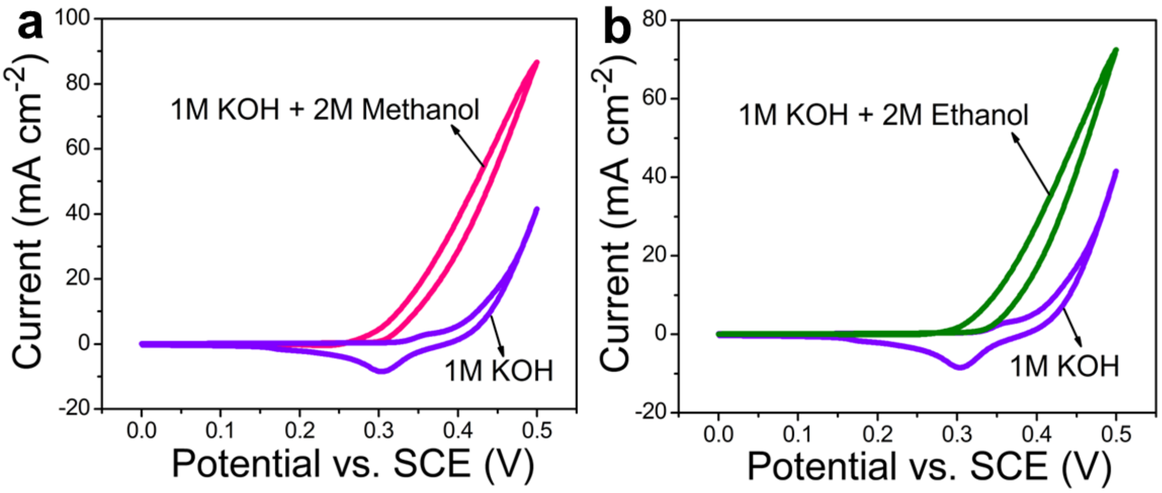

Fig. S14 CV curses of NiFe-LDH in 1 M KOH with the presence of (a) 2 M of methanol and (b) 2 M of ethanol.

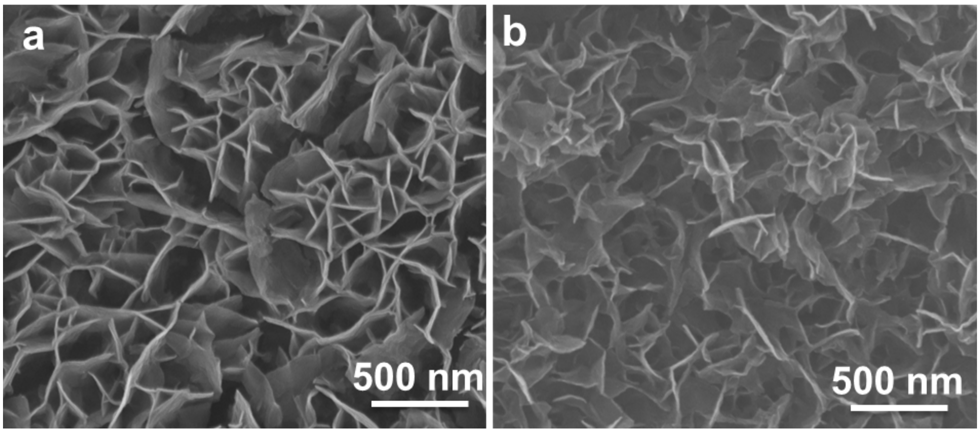

Fig. S15 SEM images of NiFe-LDH nanoplatelets arrays after the following tests: (a) OER test for 20 h; (b) hydrazine oxidation test for 5 h.

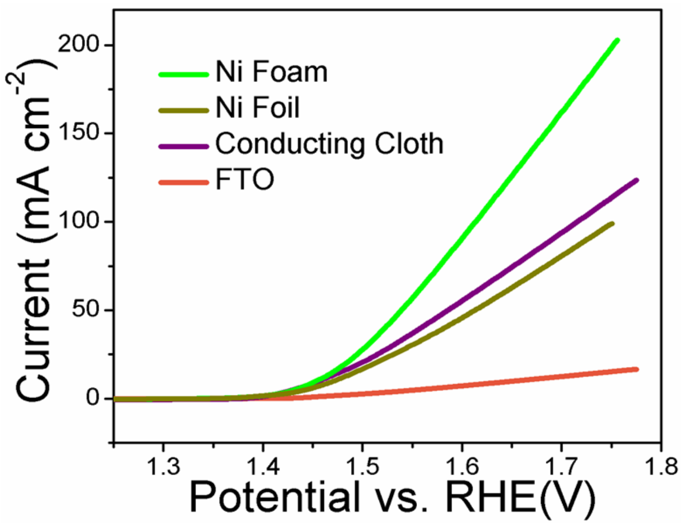

Fig. S16 Linear sweep voltammetric (LSV) curves for NiFe-LDH nanoplatelets arrays grown on different conductive substrates.

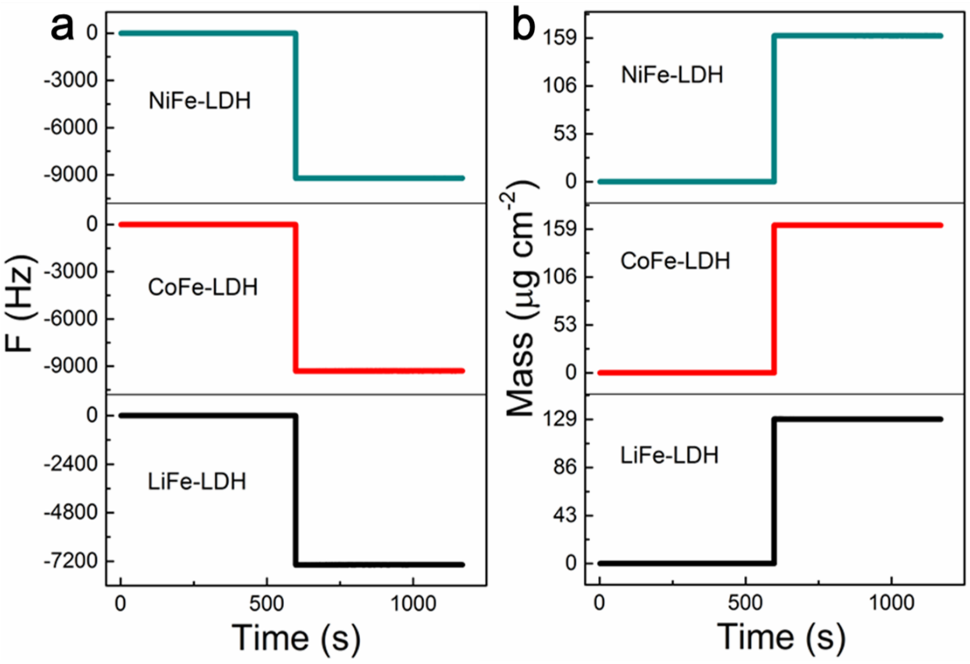

Fig. S17 (a) The resonance frequency (Δf) change curve and (b) the corresponding mass change curve in per unit area (Δm) for the gold-coated quartz crystal before and after deposited MFe-LDH (M= Ni, Co and Li).

3. Reference

1 J. Lee, K. Liang, Y. Lee, *Synthesis Met.*, 2005, **150**, 153.

2 Z. Wang, S. Xiao, Z. Zhu, X. Long, X. Zheng, X. Lu, S. Yang, *ACS Appl. Mater. Interfaces.*, 2015, **7**, 4048.

3 C. Tang, H. Wang, H. Wang, Q. Zhang, G. Tian, J. Nie, F. Wei, *Adv. Mater.*, 2015, DOI: 10.1002/adma.201501901.

4 X. Long, J. Li, S. Xiao, K. Yan, Z. Wang, H. Chen, S. Yang, *Angew. Chem. Int. Ed.*, 2014, **53**, 7584.

5 D. Tang, J. Liu, X. Wu, R. Liu, X. Han, Y. Han, H. Huang, Y. Liu, Z. Kang, *ACS. Appl.Mater. Inerfaces.*, 2014, **6**, 7918.
